# Will the negative psychological perceptions of investors reduce platform liquidity? Evidence from China’s online loans

**DOI:** 10.1371/journal.pone.0292158

**Published:** 2023-10-10

**Authors:** Zhilong Qin, Tao Liu, Xingjin Yu, Lin Yang

**Affiliations:** 1 Institute of Western China Economic Research, Southwestern University of Finance and Economics, Chengdu, Sichuan, China; 2 Gregory and Paula Chow Center for Economic Research, Xiamen University, Xiamen, Fujian, China; 3 School of Statistics, Southwestern University of Finance and Economics, Chengdu, Sichuan, China; University of Rome Tor Vergata: Universita degli Studi di Roma Tor Vergata, ITALY

## Abstract

Market liquidity can reflect whether financial market conditions are favorable and is the primary concern for investors when making investment decisions. Therefore, investors’ psychological perception and confidence in the quality of products (assets) are particularly important. Using 264 of China’s online loan platforms from August 2017 to November 2018, we investigate the impact of the negative psychological perceptions of investors on platform liquidity. The empirical results suggest that the negative psychological perceptions of investors reduce platform liquidity and increase platform liquidity risk. Using the Baidu Search Index to measure investor sentiment, we find that the negative psychological perceptions of investors affect platform liquidity by affecting investor sentiment, which provides a good channel for explaining the main conclusions. Heterogeneity analysis shows that the impact of the negative psychological perceptions of investors on platform liquidity is smaller in high-quality platforms with higher market share and higher registered capital. Meanwhile, we also find that the impact of negative psychological perceptions of investors is greater in private platforms, after the rectification policy, with positive net inflow, and in first- and second-tier cities and coastal cities. Precautionary financial regulatory policies are necessary, not punishment ex post. The research findings of this article can assist investors, platform managers, and regulatory agencies in identifying the liquidity characteristics of platforms, which can contribute to strengthening market liquidity management and financial risk control and provide some reference and support for formulating sustainable development policies in the financial industry.

## 1. Introduction

Investors’ psychological perceptions offer crucial insights to the market. Negative news or public sentiment surrounding a product can generate strong negative psychological perceptions among investors, potentially triggering panic and leading to significant fluctuations in the financial market. As a result, research on negative news or negative psychological perceptions among investors becomes particularly important. In recent years, especially in the Peer-to-Peer (P2P) market, phenomena such as running away, fraud, and illegal fundraising have brought great impact and destruction to the healthy development of the capital market and the wealth of investors, which have received widespread attention from the government, regulators, investors, and academia.

**Fig 1:**
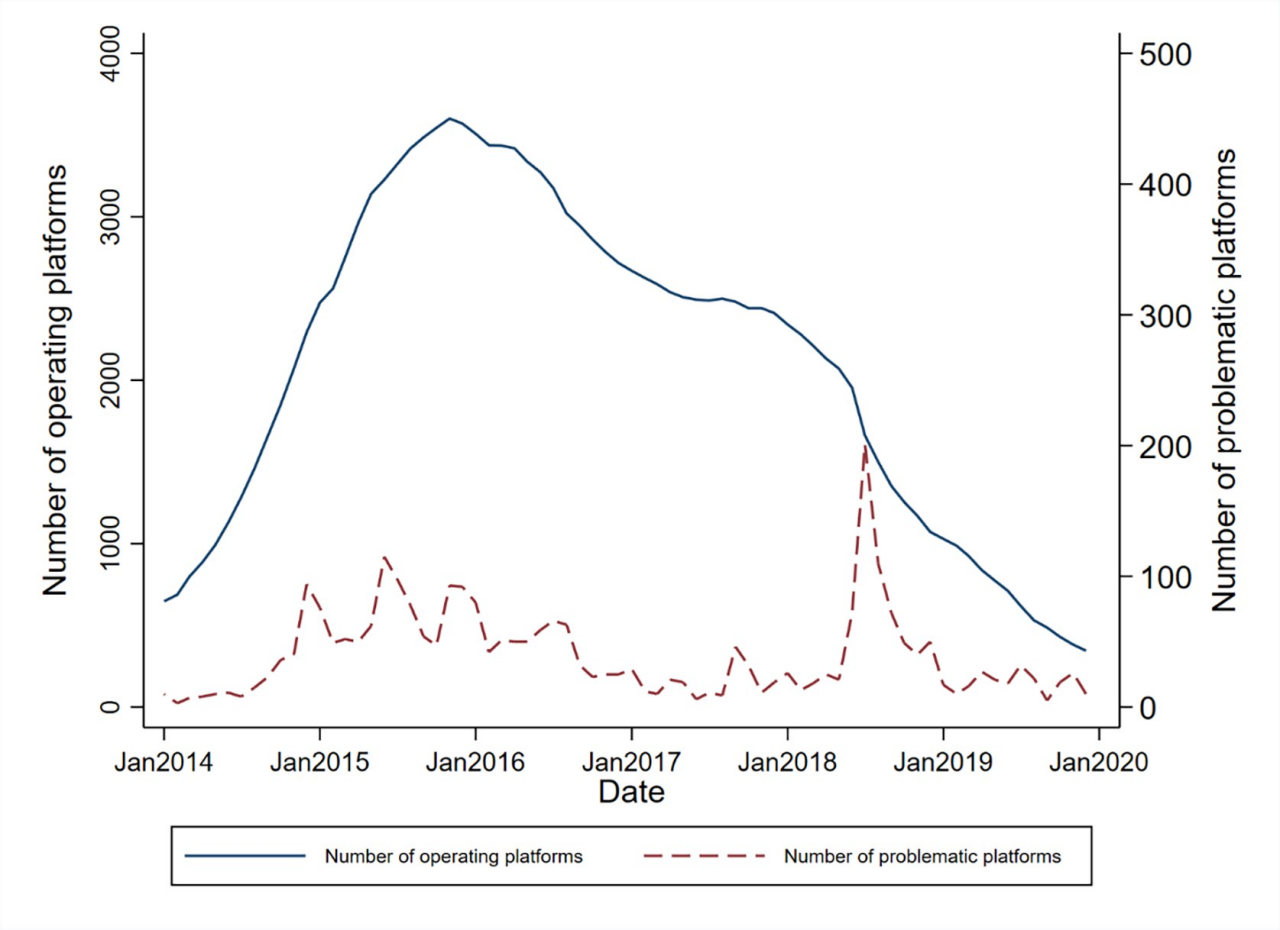
Number of operating and problematic platforms. Data source: https://www.wdzj.com/.

Due to the absence of effective policies to regulate P2P and its relatively low entry barriers, the P2P industry has experienced a high-growth period accompanied by a substantial number of problematic platforms. As shown in Fig 1, the number of P2P operating platforms in China has been in an inverse U-shape since 2014. From January 2014 to November 2015, the number of operating platforms increased from 649 to 3,579, while the cumulative number of problematic platforms was as high as 2,003 from 2015 to 2017. Since then, as P2P industry regulatory policies have been implemented and the industry’s access threshold has increased, the number of new entry platforms has decreased, and existing problematic platforms have continued to exit, leading to a monthly decrease in the number of operating platforms. As of December 2019, the number of operating platforms was only 345. At the same time, we can see that problematic platforms emerge endlessly from 2015 and 2018, and there were 200 problematic platforms a month at most. Based on the operational situation of the Chinese P2P market, it is possible to gain insights into the real challenges that China’s internet finance sector may face and the corresponding countermeasures. Simultaneously, this can also provide experiential support for other developing countries.

As an alternative to bank loans [1], Chinese P2P platforms share similarities with private equity funds (money market mutual funds), hence making liquidity a crucial aspect for these platforms. Unlike commodity sales, decentralized encroachment outperforms centralized encroachment for both suppliers and retailers [2]. Additionally, excessive fragmentation within the financial industry hampers effective supervision. Boudt and Petitjean [3] discover that news releases stimulate liquidity. Moreover, due to a wide array of psychological phenomena, negative news exerts a greater impact than positive news [4]. Most research focuses on the implications of negative news for industry development and economic behavior. An event study conducted by Bowen et al. [5] reveal that detrimental industry events heighten public concern and lead to increased industry regulatory efforts.

There is limited exploration into the effects of negative news and investors’ negative psychological perceptions on liquidity. Cahan et al. [6] discuss the influence of news content and quality on corporate liquidity. Dayanandan et al. [7] examine the impact of profit warnings on market liquidity using data from U.S. listed companies and find that voluntary disclosure of negative news improved market liquidity. The existence of Corporate Hypocrisy behavior often leads to corporate social responsibility (CSR) reports contradicting the stated CSR standards. Companies deploy various strategies to amplify information asymmetry and mitigate the impact of negative news, thereby hindering accurate psychological perception among investors. Consequently, studying negative news or investors’ negative psychological perceptions from a corporate perspective has limitations. However how negative news or negative psychological perceptions among investors affect the financial market is much of importance, to fill this gap, this paper leverages the unique attributes of the P2P market that negative news about problematic platforms is difficult to conceal, making it more representative and persuasive for investigating the impact of investors’ negative psychological perceptions on liquidity based on this market.

How do these problematic platforms affect the operation of the industry? Li and Shen [8] point out that during periods of risk events, overall market sentiment declines, and investors are more cautious about online lending investments. Both normal platforms and problematic platforms with potential risks are affected. Especially in the internet era, supervision and attention from public and media reports and spontaneous dissemination by investors have increased the transparency of P2P market information and reduced the information asymmetry between lenders and platforms. Therefore, investors develop negative psychological perceptions of problematic platforms, which has an impact on both investors and the overall sector.

In this paper, we investigate whether the negative psychological perceptions of investors reduce the platform liquidity of a special fintech market using Chinese P2P online lending platforms. Using a novel database from Wind Financial Terminal (WFT), WDZJ (www.wdzj.com), and some manually collected data, our sample ultimately includes 264 P2P lending platforms in eight provinces from August 2017 to November 2018. We find consistent evidence that the negative psychological perceptions of investors reduce platform liquidity. Furthermore, we also find that the liquidity of higher-quality platforms suffers less impact from the negative psychological perceptions of investors, while the liquidity levels of platforms with different backgrounds and registered capital do not suffer different impacts when negative news occurs.

This paper offers several potential contributions to the field of behavioral finance, particularly within the P2P market domain. First, this paper integrates behavioral finance theory into the P2P market by examining the influence of investors’ negative psychological perceptions on platform liquidity. This approach enhances the existing research on information disclosure and the effect of negative news. By examining investors’ negative psychological perceptions in the P2P market, it becomes possible to circumvent Corporate Hypocrisy behavior, rendering the discussion of the impact of investors’ negative psychological perceptions on liquidity more representative and persuasive. Second, this paper discusses investors’ negative psychological perceptions from the perspective of P2P platforms, with the goal of attaining a precise research identification. Studying from the perspective of P2P platforms can mitigate potential interference from corporate-level data that may implicitly contain Corporate Hypocrisy behavior. Due to the existence of such behavior, companies employ diverse strategies to increase information asymmetry and reduce the impact of negative news, thereby impeding accurate psychological perception among investors. Drawing on the existing research on P2P market participant characteristics [9, 10], P2P lending rates [11], and methods to reduce information asymmetry [12], this paper utilizes microlevel data from Chinese P2P platforms to investigate a pivotal issue in the online lending field: how investors’ negative psychological perceptions impact platform liquidity, making it possible to avoid the disruption caused by Corporate Hypocrisy behavior and attain a precise research identification. Lastly, investor sentiment is one critical mechanism behind the herd behavior of investors. While previous research faced challenges in effectively measuring investor sentiment due to data constraints, the advancement of big data has opened new avenues. It’s now feasible to quantify investor behavior through metrics such as search engine data, leading to the creation of more precise investor sentiment indicators [13–16]. This paper innovatively employs the Baidu Search Index to depict investor sentiment in P2P market, offering fresh and novel insights into the investigation of the mechanisms underlying herd behavior among investors.

The rest of the paper proceeds as follows. Section 2 presents the research hypotheses. Section 3 describes the data and model specification. Section 4 presents the empirical results. Section 5 further reports the channel analysis. Section 6 concludes and provides policy implications.

## 2. Research hypotheses

### 2.1. Negative psychological perceptions of investors and platform liquidity

Due to a wide range of psychological phenomena, negative news tends to exert a stronger influence on investors compared to positive news [4]. Moreover, most of the studies on the negative psychological perceptions of investors and economic behavior are related to the stock market and firm behavior, and event studies have found significant negative effects of malevolent industry events on the industry [5, 17]. While the impact direction of negative psychological perceptions of investors on market liquidity is uncertain. Some studies suggest that the negative psychological perceptions of investors increase market liquidity [3], whereas others show that the negative psychological perceptions of investors decrease market liquidity [6, 7].

The P2P industry is controversial and is frequently accompanied by negative reports such as runs, illegal fundraising, and online fraud. Liu and Ye [18] categorize search volume into news-driven and self-initiated (active search) to reveal the investment behavior of individual/retail investors. Their findings illustrate that news reports influence search behavior and subsequently impact the stock market. A cluster of problems, such as runs and collapses of P2P platform, can result in negative psychological perceptions among investors, thereby affecting the industry. Notably, investor behavior in the online lending market exhibits a herding effect [19]. Consequently, negative news surrounding a single platform can incite a wave of negative psychological perceptions among investors. This can significantly dent investors’ confidence, triggering a cascade effect where multiple investors exit the P2P industry. Consequently, the platform might suffer a liquidity crisis due to substantial capital outflows, which eventually leads to a run on the entire P2P industry. Accordingly, this paper proposes Hypothesis 1.

**Hypothesis 1**: The negative psychological perceptions of investors can reduce platform liquidity and increase platform liquidity risk.

### 2.2. The impact of platform background and quality

Categorically, based on the primary shareholders, P2P online lending platforms in China can be categorized into the private sector, banking sector, listed sector, state-owned sector, and venture capital sector. Notably, Jiang et al. [20] discover that government involvement serves as an effective signal for investors. P2P platforms associated with state-owned enterprises (SoEs) exhibit higher trading volumes, attract a larger investor base, and even display better resilience, particularly during market downturns. Wang and Zou [21] suggest that the influences of platform characteristics on trading volume differ across various platform types. Consequently, it becomes imperative to ascertain the factors influencing trading volume in disparate background platforms during platform selection. Moreover, Wang and Zhang [22] demonstrate that, when compared to platforms with private major shareholders, platforms associated with venture capital, listed companies, and state-owned assets have a reduced likelihood of encountering problematic situations by 10.8%, 8.8%, and 13.2%, respectively. Platform background, as an endorsement of platform credibility, can to some extent accommodate or balance investors’ negative psychological perceptions. Therefore, the effects of investors’ negative psychological perceptions might differ among various platform types, and platforms with stronger backgrounds experience less pronounced liquidity impacts.

We evaluate platform quality from the two perspectives of registered capital and market share. In general, platforms with higher registered capital and market share tend to exhibit better quality. In the context of P2P platforms, the registered capital, especially the paid-in capital, embodies the platform’s invested capital, forming the bedrock for its smooth functioning. This capital figure indicates the platform’s robustness and its ability to weather adverse conditions, such as the occurrence of bad debts. In a broad sense, a higher registered capital correlates with a higher credibility of the platform. While the absolute value of registered capital might not precisely indicate the platform’s specific strength, it certainly contributes to the operational capabilities of the platform and its capacity to manage bad debts during subsequent phases of operation. Notably, Wang and Zhang [22] disclose that for each 1% increment in registered capital, the likelihood of a platform being problematic diminishes by 2.6%.

Furthermore, during financial crises, investors tend to reallocate their assets toward more liquid options. Platforms possessing higher levels of liquid assets experience less credit risk and better asset quality. Similarly, platforms with greater market share exhibit improved payment capacity and heightened quality. In 2018, the regulatory agency required P2P platforms to file for the record, and some problematic platforms continued to emerge, which was followed by a “thunder wave” of the P2P industry. Since then, surviving platforms have met the needs of the market, but the market share of each platform still varies greatly. According to the public data of Wangdaizhijia, as of March 2019, the turnover of the top 10% and 30% of platforms in the P2P industry accounted for approximately 50% and 70% of the total turnover of the whole P2P industry, respectively. The P2P industry presents an obvious “Matthew Effect” by which platforms with good quality become increasingly strong and the trading volume increases, but those with poor quality worsen and the turnover decreases. Some studies have shown that investors have a “flight to high quality” [23].

As the foundation of a platform’s normal operations and a gauge of platform credit risk, platform quality can to some extent accommodate or balance investors’ negative psychological perceptions. Building on the above analysis, the quality of a P2P platform, represented by its registered capital and market share, can indeed exert an influence on trading volume. Accordingly, this leads to the formulation of Hypothesis 2 as follows.

**Hypothesis 2**: The variation in platform backgrounds and quality results in significant heterogeneity in the impact of investors’ negative psychological perceptions on platform liquidity. This variation underscores the notion that investors can adapt or offset their negative psychological perceptions through consideration of the platform’s background and quality.

### 2.3. The channel role of investor sentiment

The development of big data provides previously unavailable measures of investor behavior, the frequency of internet searches for stocks has been widely used to measure investor attention, and Google (Baidu) is representative of the internet search behavior of the general population in the United States (China). Therefore, measuring attention based on search engine data and thus constructing investor sentiment indicators has become increasingly popular among researchers. Google search volume is commonly adopted to measure the investor attention of foreign financial markets [13, 14], while the Baidu Search Index is commonly adopted to measure the investor attention of domestic financial markets [15, 16]. Relevant studies have shown that investor attention measured by the Baidu Search Index can effectively explain stock market liquidity [18, 24]. When sentiment is optimistic, stock markets are highly liquid and vice versa [25, 26].

In addition, Yang and Mo [27] discover that news and social media affect investors’ sentiments, which drives the upward or downward movements of financial markets. Investor sentiment refers to systematic biases in investors’ future expectations [28]. While traditional financial theory asserts that stock prices are the present value of expected future profits for listed companies, the formation of stock prices in actual trading processes also heavily depends on investor sentiment. Similarly, within the online lending market, the valuation and reputation of P2P companies, akin to the stock prices of listed firms, rely on investor sentiment. It is widely acknowledged that customer perception encompasses the impressions, cognition, and/or awareness that customers hold about a company or its products. Customer perception plays an integral role in a company’s ability to attract new customers and retain existing ones, and it is influenced by factors such as advertising and marketing strategies [29]. In financial markets, investors, much like consumers, possess the ability to perceive financial products. Hence, investor psychological perception can be understood as the psychological awareness investors develop while engaging in the consumption process. Consequently, we posit that a logical chain exists within the P2P market: negative news (investor’s negative psychological perception) → investor sentiment → platform trading volume (platform liquidity). Accordingly, this paper proposes Hypothesis 3 as follows.

**Hypothesis 3**: Negative psychological perceptions of investors impact platform liquidity through investor sentiment.

## 3. Empirical design

### 3.1. Data collection

To explore the influence and underlying mechanism of investors’ negative psychological perceptions on platform liquidity and the associated liquidity risk. This paper collects platform data from the WFT (Wind Financial Terminal) and WDZJ (www.wdzj.com). The WFT contains the most complete data and information on China’s financial market and provides systematic data on China’s P2P platforms, including platform trading volume, average rate of return of all bid projects on the platform, outstanding funds on the platform, and so on. We also control for macroeconomic variables, which comprise the Shanghai Interbank Offered Rate (Shibor), per capita GDP, and GDP-related savings. These data are sourced from WFT. Additionally, WDZJ is a publicly accessible website committed to disclosing information about internet finance within China. The data related to problematic platforms in this article originate from WDZJ. Furthermore, it provides comprehensive insights into China’s P2P platforms, encompassing their background, registered capital, and registration time. As the WDZJ provides the number of problematic platforms in the eight provinces (Beijing, Shanghai, Sichuan, Shandong, Guangdong, Jiangsu, Zhejiang and Hubei) with most platforms, the platforms considered in this paper consist of 264 P2P lending platforms from these eight provinces, spanning from August 2017 to November 2018.

In this paper, we have implemented specific measures to address the challenges posed by outliers and missing values within our sample. Firstly, to maintain the integrity of our estimation results, we have decided to discard samples that have a transaction volume of 0. This is primarily because taking the logarithm of a value with 0 transaction volume is inherently meaningless. Furthermore, when considering the negative psychological perceptions of investors, or the number of problematic platforms, it’s crucial to recognize that these influence the platform liquidity, or the platform trading volume, of the subsequent period. Given our data structure, we’ve opted to lag other variables by one period. The only exception to this is the platform transaction volume data, which remains based on current data. As a result of these decisions, our finalized sample consists of 3,287 observations.

### 3.2. Variable definitions

#### 3.2.1. Platform liquidity

Drawing on the research of Kyle [30] and Pagano [31], where they utilize trading volume as a proxy variable for market liquidity, which effectively captures market liquidity changes and reflects investors’ market judgments, they discover that the trading intensity of informed investors is positively correlated with market liquidity. Consequently, this paper adopts platform transaction volume as an indicator of platform liquidity, which is measured by the total investment absorbed by the platform within a specific period.

#### 3.2.2. Problematic platform

Through event studies, significant negative effects of adverse events on the industry can be discovered [32]. When an illegal fundraising P2P platform defaults and absconds, it triggers public “panic”. Existing investors withdraw funds, and new investors hesitate to enter, disrupting the equilibrium of existing platforms and potentially leading to industry-wide defaults. Additionally, problematic platforms often attract media coverage, serving as negative news impacting the industry. Therefore, the number of problematic platforms serves as a representative indicator to measure the severity of negative news, which will also cause investors to have a negative psychological perception of the P2P industry. This paper adopts the number of problematic platforms as an indicator of negative psychological perceptions of investors in the P2P industry.

Investor behavior always tends to have local bias [33]. When problematic platforms emerge in a particular province, it inevitably leads to an excessive focus by investors on the industry, resulting in reduced investments in local platforms and subsequently causing a decline in transaction volumes for these local platforms. We also find that the current number of problematic platforms arises from the previous period’s operating platforms. To avoid overestimating the impact of negative news on platform liquidity and precisely capturing the extent of negative psychological perception, this paper excludes the number of problematic platforms in the focal province and mainly takes the proportion of the number of problematic platforms in other provinces over the number of operating platforms in other provinces in the last period as the main explanatory variable. That is, the proportion of problematic platforms in Beijing in one period is the total number of problematic platforms in other provinces except Beijing in the period divided by the total number of operating platforms in other provinces except Beijing in the last period. Furthermore, for simplicity, we lag the number of operating platforms in other provinces by one period to mitigate any potential endogeneity issues stemming from contemporaneous measurement concerns between our dependent variables and problematic platforms.

#### 3.2.3. Investor sentiment

Given that the negative psychological perceptions of investors might influence platform liquidity through their investor sentiment, this paper draws inspiration from relevant research on “limited attention” in stock markets and employs the Baidu Search Index as a measure of “limited attention” among P2P participants [15, 16]. Given the significance of “running away” and “thunder wave” as critical events in the P2P industry, this paper utilizes the sum of the Baidu Search Index for the keywords “P2P running away” and “P2P thunder wave” in each province to reflect investors’ attention toward problematic platforms, serving as a good proxy variable for investor sentiment.

#### 3.2.4. Control variables

To mitigate potential estimation bias resulting from omitted variable concerns, this paper also incorporates control variables, including platform characteristics and provincial macroeconomic features. The control variables of platform characteristics include the average rate of return of all bid projects on the platform (Rate), which are expected to have a positive impact on platform liquidity since a higher rate of return reflects the gain from investing in platforms. The higher the interest rate is, the greater the attraction for investors, leading to larger platform transaction volumes. However, with risks accompanying returns, investors are rational and prudent. When interest rates are excessively high, investors become cautious, which can lead to a no significant impact of interest rates on liquidity. We also control for the sum of all outstanding funds on the platform (Balance), which measures the size of the platform. Generally, larger platforms are more likely to attract investors’ favor and tend to have higher liquidity. Last, we incorporate controls for platform age in months (Age) measured by sample time minus registration time, as well as the maturity of the platform (Maturity), defined as the average loan period (months) for all projects on the platform. As the registration time of a platform increases, the platform’s customer base tends to become more stable, which could lead to poorer liquidity. Similarly, when the average loan period increases, indicating longer borrower terms of projects and less attractiveness for investors, the platform’s liquidity often deteriorates. That is, both the age and maturity of the platform are expected to have a negative impact on platform liquidity.

For provincial macroeconomic features, we mainly incorporate the Shibor, which reflects the central bank’s monetary policy. A higher value of Shibor means a higher borrowing cost and lower likelihood from banks, resulting in the demand for funds shifting toward P2P platforms and an increase in platform transaction volume. Finally, we incorporate per capita GDP and GDP-related savings to capture the economic development and financial development of provinces, which are expected to have a positive impact on platform liquidity because when the level of economic development and financial development of a province is higher, regional investment activities are more active, so the demand for funds may be higher, and the liquidity of P2P platforms may be stronger. However, this impact may not be significant for P2P platform liquidity due to the higher level of development of financial institutions.

Table 1 provides the definitions of the variables and the data sources, while Table 2 reports the descriptive statistics of these variables.

**Table 1:** Variable definitions and data sources.

Variables	Definitions	Sources
Liquidity	Logarithm of the transaction volume (ten thousand yuan), that is, the total amount of investment absorbed by the platform in a certain period of time.	WFT
Problematic platform	Log of the number of problematic platforms in other provinces over the number of operating platforms in other provinces*100.	WDZJ
Rate	The average rate of return of all bid projects on the platform.	WFT
Balance	Logarithm of the sum of all outstanding funds on the platform.	WFT
Age	Logarithm of platform age (months), sample time minus online time.	WDZJ
Maturity	Logarithm of the average loan period (months) for all projects on the platform.	WFT
Shibor	Shanghai Interbank Offered Rate (Shibor): 1 month.	WFT
Savings per unit of GDP	Logarithm of the financial institution deposit balance per unit of GDP, that is, the financial institution deposit balance divided by GDP.	WFT
GDP per capita	Logarithm of per capita GDP, that is, GDP divided by the number of permanent residents.	WFT

**Table 2:** Descriptive statistics.

Variables	N	Mean	SD	Min	Max
Liquidity	3,287	8.215	1.530	-3.912	11.920
Problematic platform	3,287	1.747	2.321	0.189	11.118
Rate	3,287	10.791	2.447	4.960	21.460
Balance	3,287	9.685	1.479	1.959	13.613
Age	3,287	3.576	0.343	0.693	4.443
Maturity	3,287	1.438	0.755	-2.526	3.804
Shibor	3,287	3.886	0.567	2.689	4.935
Saving per unit of GDP	3,287	2.970	0.378	2.297	3.870
GDP per capita	3,287	8.990	0.312	8.139	9.520

### 3.3. Model specification

Consistent with Li et al. [2], we adopt the dual fixed effects model as the identification strategy model to identify the impact of the negative psychological perceptions of investors on platform liquidity, as shown below:

$${Liquidity}_{i,p,t}=\alpha +\beta {Problematic\;platform}_{i,p,t-1}+{\gamma X}_{i,p,t-1}+{\varphi Z}_{i,p,t-1}+\lambda_{i}+\delta_{t}{+\varepsilon }_{i,p,t}$$ (1)

where *i*, *p*, and *t* are subscripts representing the platform, province, and month, respectively. *Liquidityi*_*i*,*p*,*t*_ is the logarithm of the transaction volume, which measures platform liquidity, as transaction volume can well measure changes in platform liquidity and reflect investors’ judgments on the market [31]. *Problematic platform*_*i*,*p*,*t*−1_ is the key explanatory variable that measures the negative psychological perceptions of investors in the P2P industry. Problematic platforms usually lead to reports from the relevant media producing negative psychological perceptions of investors, which affect the industry. Therefore, the number of problematic platforms can be a proxy variable to measure the severity of the negative psychological perceptions of investors. *X*_*i*,*p*,*t*−1_ are the platform-level control variables, including the average rate of return of all bid projects, outstanding funds, the platform’s age and the average loan period (months) for all projects. *Z*_*i*,*p*,*t*−1_ are the provincial macrolevel control variables, including savings per GDP and GDP per capita. All these variables are defined in Table 1. Accordingly, *β*, γ and *φ* are the coefficients reflecting the effects on volume. *λ*_*i*_ is the platform fixed effect, *δ*_*t*_ is the time fixed effect, and *ε*_*i*,*p*,*t*_ is the random error term clustered at the platform level. *α* is the intercept term.

## 4. Empirical results

### 4.1. Baseline results

Table 3 presents the regression results for the impact of negative psychological perceptions of investors on transaction volume. Notably, in Column (1), the results illustrate a significant negative effect of problematic platforms on transaction volume. Upon the introduction of additional control variables, as depicted in Columns (2) to (3), the results remain robust, indicating that when the negative psychological perceptions of investors increase, platform transaction volume decreases significantly. This substantiates the assertion that negative psychological perceptions of investors reduce platform liquidity and increase platform liquidity risk. Specifically, as shown in Column (1) of Table 3, without adding any control variables, a 1% increase in the negative psychological perceptions of investors (problematic platform) leads to a 0.156% decrease in platform transaction volume, which is significant at the 1% confidence level.

**Table 3:** Regression results of the negative psychological perceptions of investors on liquidity.

Variables	Liquidity		
	(1)	(2)	(3)
Problematic platform	-0.156***	-0.034**	-0.036***
	(0.015)	(0.013)	(0.013)
Rate		0.034	0.034
		(0.022)	(0.021)
Balance		0.748***	0.730***
		(0.092)	(0.094)
Age		-0.633	-0.675
		(0.478)	(0.479)
Maturity		-0.316***	-0.320***
		(0.084)	(0.085)
Shibor			0.289**
			(0.115)
Saving per unit of GDP			0.090
			(1.957)
GDP per capita			0.101
			(1.907)
Platform/Time	YES	YES	YES
Observations	3287	3287	3287
*R* ^2^	0.819	0.851	0.852

Note: We adopt the method of gradually adding control variables to test the impact of the negative psychological perceptions of investors on platform trading volume. Column (1) adds only indicators of the negative psychological perceptions of investors, and columns (2) and (3) sequentially add platform characteristic variables and provincial-level macroeconomic characteristic variables. The results remain stable.

***, **, and * are significant at the 1%, 5%, and 10% levels, respectively, and the robust standard errors for clustering at the platform level are in parentheses.

Furthermore, as shown in column (2) of Table 3, we add the platform characteristics variables, and the results show that the effect of pending balance on platform transaction volume is significantly positive, which is consistent with Jiang [34]. This could be attributed to the pending balance measures the development potential of the platform to a certain extent; thus, a higher pending balance indicates that the platform has more loan business and higher turnover. Additionally, there is a significant negative correlation between the average borrowing period and platform turnover, which is consistent with the findings of Chen et al. [35]. Conversely, the interest rate and age of the platform have no significant effect on platform transaction volume.

Column (3) includes macroeconomic characteristic variables, and the results show that when the negative psychological perceptions of investors (problematic platform) increase by 1%, the platform transaction volume significantly decreases by 0.036% at the 1% level. Additionally, the Shanghai Interbank Offered Rate (Shibor), an indicator of the central bank’s monetary policy, demonstrates a substantial positive influence on platform transaction volume. A rise in Shibor signals that there is a shortage of funds in the market, it is more expensive to borrow money from banks, i.e., the availability of funds is lower, demanders of funds flow to online lending platforms, and the volume of platform transactions increases. However, GDP per capita and savings per unit GDP have positive but not significant impacts on platform liquidity.

Our findings are consistent with the observed herding behavior of investors in the context of online P2P lending [36]. When negative news is issued, it is not that one investor will choose to withdraw from the P2P industry, but the confidence of all investors will be affected. Consequently, many investors choose to withdraw from the P2P industry. Therefore, the large-scale escape of funds will cause the platform to encounter a liquidity crisis, which will cause a run on the entire industry [37–39]. Therefore, the research results verify hypothesis H1.

### 4.2. Robustness tests

To illustrate the robustness of our findings, we conduct a series of robustness tests, and the results are shown in Table 4. All results are robust to the baseline estimation. First, we change the cluster group of the standard errors. In the baseline, the standard errors are clustered at the platform level and by year and month. The previous analyses indicate that the impacts of negative psychological perceptions of investors on platform liquidity may differ across city types and the distribution of platforms by province. Thus, in Column (1) of Table 4, we modify the cluster group into city, year and month. The results demonstrate that the negative psychological perceptions of investors had a significantly negative impact on platform liquidity, reaffirming the consistency of our baseline conclusions.

**Table 4:** Robustness check: Adjust the cluster, FE, and sample size and consider rectification policies and quantile narrowing.

	(1)	(2)	(3)	(4)	(5)
	Cluster	Province by year-month	Quantity>10	Policy	Winsorized
Problematic platform	-0.035***	-0.035***	-0.044***	-0.057***	-0.028***
	(0.007)	(0.013)	(0.017)	(0.010)	(0.010)
Control variables	YES	YES	YES	YES	YES
Platform/Time	YES	YES	YES	YES	YES
Observations	3287	3287	2419	3287	3,287
*R* ^2^	0.857	0.853	0.836	0.850	0.879

Note

***, **, and * are significant at the 1%, 5%, and 10% levels, respectively, and the robust standard errors for clustering at the platform level are in parentheses, except for Column (1).

Since there may be some provincial factors other than the macroeconomic characteristic variables for which we control that may affect the development of the P2P platform in different provinces, we add province-by-year fixed effects in Column (2) of Table 4. By doing so, we avoid the endogeneity problems caused by possible omitted variables, and the result remains consistent with the baseline estimation.

We consider that the outliers may be caused by too few platforms in different cities and further cause estimation errors. We focus on samples whose cities have more than ten platforms. As the number of platforms increases in the same city, the effect of the negative psychological perceptions of investors on platform liquidity may increase, as Column (3) shows. The estimation results and baseline estimation exhibit no significant difference, so our estimation results are relatively stable. Since 2017, supervision of the P2P industry has been restricted because of problematic platforms. To exclude the policy impacts, we add policy variables (which are equal to 1 after the rectification policy and 0 otherwise). The result in Column (4) shows that the decrease in platform liquidity is mainly caused by the negative psychological perceptions of investors.

Furthermore, to eliminate the influence of sample outliers, we conduct 1% and 99% quantile narrowing of the transaction volume. The result remains indifferent between the estimation result and the baseline estimation, so the result of our estimation is relatively stable.

Considering the geographical and economic disparities between provinces, this paper employs an economic geography matrix to compute the proportion of problematic platforms and replace the original explanatory variable with this proportion in the regression analysis. The results are presented in Column (1) of Table 5. Additionally, the number of borrowers is utilized as a metric for platform liquidity since, given the negative psychological perceptions of investors, borrowers might worry about the negative impact on their creditworthiness, which further affects platform liquidity. Notably, while customers exhibiting loyalty tendencies might exhibit greater tolerance toward pricing fluctuations [40], they tend to be more sensitive to the negative psychological perceptions of investors. These findings are depicted in Column (2) of Table 5. The results in both Column (1) and Column (2) indicate that the proportion of problematic platforms can significantly reduce platform liquidity by replacing the main explanatory measurement in Column (1) and the main explained variable definition in Column (2), that is, the conclusion that the negative psychological perceptions of investors can significantly reduce platform liquidity and increase platform liquidity risk, indicating the robustness of our main conclusions.

**Table 5:** Robustness check: Adjust the explanatory variable.

	(1)	(2)
	Economic geography matrix	Borrowers
Problematic platform	-0.053***	-0.047***
	(0.010)	(0.012)
Control variables	YES	YES
Platform/Time	YES	YES
Observations	3,287	3,287
*R* ^2^	0.326	0.051

Note

***, **, and * are significant at the 1%, 5%, and 10% levels, respectively, and the robust standard errors for clustering at the platform level are in parentheses. The same applies below.

### 4.3. Heterogeneous effects

**Fig 2:**
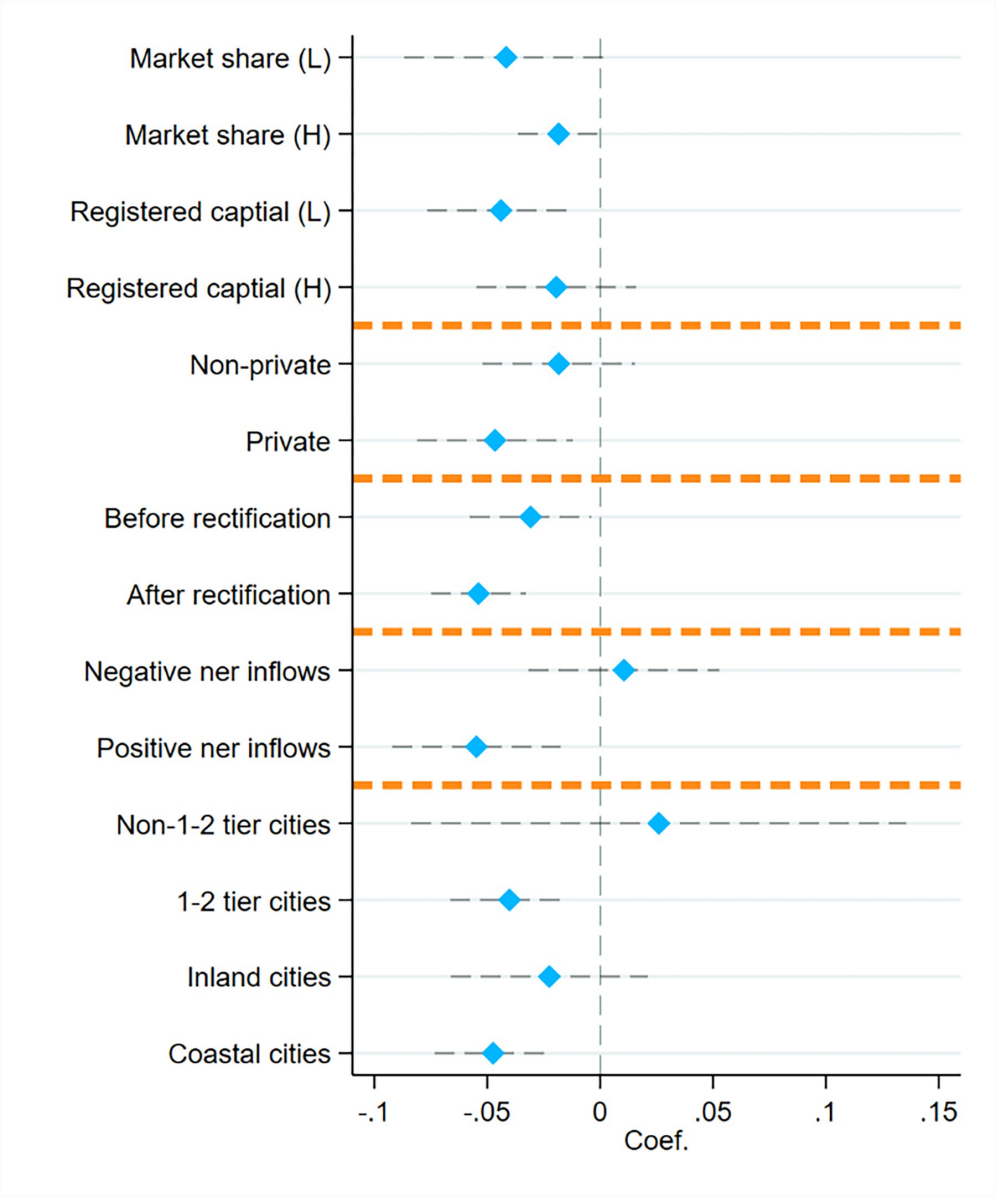
Heterogeneous effects of the negative psychological perceptions of investors on platform liquidity. Note: Blue diamond’s mark the estimated coefficients, and the dashed black lines show 95% confidence intervals. Each row corresponds to a separate regression using a corresponding subsample. We adopt the mean values to separate the high (H) from the low (L) group for the first two pairs of heterogeneity analyses. For example, if a platform’s market share is higher than its mean market share, the platform falls into a high market share group. For platform type, the sample is divided into private or non-private groups. The dashed orange lines divide our heterogeneity analyses into five categories (from top to bottom): platform quality, platform background, rectification policy, net inflows, and city characteristics. Each regression implements the first model (Eq (1)) and controls for all control variables and fixed effects.

Fig 2 shows the heterogeneity analysis results. It shows that the effects of the negative psychological perceptions of investors on platform liquidity are greater on platforms with lower market share, lower registered capital and smaller size. Not surprisingly, these results confirm that the negative psychological perceptions of investors have much larger effects on these platforms because they tend to be less credible. These results are consistent with the findings of Brunnermeier and Pedersen [23] that investors exhibit the phenomenon of escaping to high quality. When investors have negative psychological perceptions, they tend to believe that platforms with a higher market share, higher registered capital and a larger size are of better quality and have less risk.

According to the nature of the main shareholders, P2P platforms in China are divided into five types: private, banking, listed, state-owned and VC (venture capital). The results show that the negative psychological perceptions of investors have greater effects on private than on non-private platforms and that these effects are significantly negative. This finding is consistent with the theory that when a platform’s background is stronger, so are its operational, strategic, and risk management capabilities, and the probability of the platform being problematic is smaller.

The above results verify H2. In addition to analyzing the heterogeneity of this paper from the perspective of platform quality and background, we also conduct heterogeneity analysis based on the impact of rectification policy, net inflow and urban characteristics.

Furthermore, this paper analyses the impact of the rectification policy and highlights that the negative psychological perceptions of investors had a larger effect on platform liquidity after rectification. This finding aligns harmoniously with investor attention theory, which states that policy can attract much more attention from investors than other efforts. Additionally, the study reveals that the negative psychological perceptions of investors exerted a more substantial impact on platforms exhibiting positive net inflows, whereas platforms with negative net inflows displayed no significant effect. This phenomenon likely stems from the fact platforms with large capital inflows may be more affected by the negative psychological perceptions of investors, whereas other platforms have already suffered capital outflows. Finally, the results show that the negative psychological perceptions of investors have a greater effect on the platforms in first- and second-tier cities as well as coastal cities, which means that investors in these cities may be more cautious about the negative psychological perceptions of other investors.

## 5. Channel analysis

To test H3, the regression analysis of investor sentiment is conducted using Model (2), and the results are shown in Table 6. The model is specified as follows:

$${Investor\;sentiment}_{i,p,t}=\alpha +\eta {Problematic\;platform}_{i,p,t-1}+{\gamma X}_{i,p,t-1}+{\varphi Z}_{i,p,t-1}+\lambda_{i}+\delta_{t}{+\varepsilon }_{i,p,t}$$ (2)

where *Investor sentiment*_*i*,*p*,*t*_ is the logarithm of the Baidu Search Index, which measures investor sentiment, and all the control variables are consistent with Model (1). Consistent with the analysis process of Model (1), we use the method of gradually adding control variables to test the impact of the negative psychological perceptions of investors on investor sentiment.

**Table 6:** Regression results of the negative psychological perceptions of investors on investor sentiment.

Variables	Investor sentiment		
	(1)	(2)	(3)
Problematic platform	0.057***	0.034***	0.106***
	(0.003)	(0.002)	(0.010)
Platform characteristic variables		YES	YES
Macroeconomic characteristic variables			YES
Platform/Time	YES	YES	YES
Observations	3287	3287	3287
*R* ^2^	0.819	0.851	0.852

Note

***, **, and * indicate significance at the 1%, 5%, and 10% levels, respectively, and the robust standard errors are in parentheses and are clustered at the platform level by year and by month. “YES” means the fixed effects are controlled for. The control variables are the same as in Table 3.

As posited by the research hypothesis, negative psychological perceptions of investors may potentially influence platform liquidity through their impact on investor sentiment. Column (1) of Table 6 controls for only platform and time fixed effects but without control variables, and its coefficient is significant at the 1% level. This finding indicates that an increase in the negative psychological perceptions of investors attracts investors’ attention, which impacts investor sentiment. Columns (2)-(3) gradually add the platform characteristics and macroeconomic characteristics variables, and the results remain robust. Thus, the negative psychological perceptions of investors have a significant positive effect on investor sentiment, and this result is robust. In line with Yang and Mo [27], who demonstrate the influence of news and social media on investors’ sentiments, this paper reveals that negative psychological perceptions induced by negative news significantly amplify investor sentiment. The results of this paper provide evidence that investor sentiment serves as a crucial mechanism through which negative psychological perceptions of investors impact platform liquidity and verify Hypothesis 3.

## 6. Conclusions

This paper investigates the impact of negative psychological perceptions of investors on platform liquidity. Empirical results show that problematic platforms, such as those that produce negative psychological perceptions of investors, reduce platform liquidity, and increase platform liquidity risk. These results are stable based on several robustness checks. By employing the Baidu Search Index to measure investor sentiment, we find that the negative psychological perceptions of investors can affect platform liquidity by influencing investor sentiment, which serves as a good channel for explaining the main conclusion. Moreover, we find that the pending balance and Shanghai interbank offered rate have significantly positive impacts on platform transaction volume, while the average borrowing period has significantly negative impacts on platform transaction volume.

Heterogenous analysis reveals that the effects of the negative psychological perceptions of investors on platform liquidity are pronounced within platforms with lower market share and registered capital. These findings provide insights into how to measure platform quality (i.e., market share and registered capital can be used as measures of platform quality). We can also conclude that the platform background can be used as a guarantee and good endorsement of platform professionalism. When the negative psychological perceptions of investors occur, investor behavior shows the characteristics of fleeing to a high-quality platform, and investors in first- and second-tier cities and coastal cities may be more cautious about the negative psychological perceptions of other investors.

The findings of this paper have some implications for investors, platform managers and regulators. First, in addition to the platform yield, investors need to pay attention to the platform’s outstanding balance, platform market share, risk control capabilities and other related characteristics. Second, platform managers can increase investor confidence by improving platform transparency. Finally, regulatory agencies should strictly supervise the authenticity and effectiveness of the information disclosed by the platforms and strictly impose restrictions on the entry and exit of platforms to prevent those platforms with the impure motivation from entering the industry.

Rapid economic growth in China is one of the most remarkable phenomena in recent years. As the world’s largest developing country, the experience of Chinese offers significant insights for other nations, particularly developing countries. Focusing on the Chinese online lending market, this study has revealed that negative psychological perceptions resulting from adverse news diminish platform liquidity and increase liquidity risk. For other developing countries, market liquidity similarly reflects the health of their financial markets. Therefore, these nations should also pay close attention to the psychological perceptions of investors.

Due to data limitations, this paper has solely explored the channel effects of investor sentiment in the mechanism analysis without delving into other potential mechanisms of how negative psychological perceptions impact liquidity. Given the anticipated growth of big data in the future, we aspire to further enrich the understanding of other potential mechanisms between negative psychological perceptions and liquidity in subsequent research.

## Supporting information

S1 Dataset (XLS)

## References

[1] Tang H. Peer-to-Peer lenders versus banks: Substitutes or complements? Review of Financial Studies, 2019;32(5):1900–1938. doi: 10.1093/rfs/hhy137

[2] Li EM, Liao L, Wang ZW, Xiang HY. Venture capital capital certification and customer response: Evidence from P2P lending platforms. Journal of Corporate Finance, 2020;60:101533. doi: 10.1016/j.jcorpfin.2019.101533

[3] Boudt K, Petitjean M. Intraday liquidity dynamics and news releases around price jumps: Evidence from the DJIA stocks. Journal of Financial Markets, 2014;17:121–149. doi: 10.1016/j.finmar.2013.05.004

[4] Baumeister RF, Bratslavsky E, Finkenauer C, Vohs KD. Bad is stronger than good. Review of general psychology, 2001;5(4):323–370. doi: 10.1037/1089-2680.5.4.323

[5] Bowen RM, Castanias RP, Daley LA. Intra-industry effects of the accident at three-mile-island. Journal of Financial and Quantitative Analysis, 1983;18(1):87–111. doi: 10.2307/2330806

[6] Cahan RH, Cahan SF, Lee T, Nguyen NH. Media content, accounting quality, and liquidity volatility. European Accounting Review, 2017;26(1):1–25. doi: 10.1080/09638180.2015.1087866

[7] Dayanandan A, Donker H, Karahan G. Do voluntary disclosures of bad news improve liquidity? North American Journal of Economics and Finance, 2017;40:16–29. doi: 10.1016/j.najef.2017.01.002

[8] Li CS, Shen Y. Information identification of risk contagion: An empirical study based on P2P lending market. Journal of Finance Research, 2018;11:98–118.

[9] Duarte J, Siegel S, Young L. Trust and credit: The role of appearance in peer-to-peer lending. Review of Financial Studies, 2012;25(8):2455–2483. doi: 10.1093/rfs/hhs071

[10] Ravina E. Love& loans: The effect of beauty and personal characteristics in credit markets. Available at SSRN 1107307, 2019. doi: 10.2139/ssrn.1107307

[11] He F, Qin SQ, Zhang XT. Investor attention and platform interest rate in Chinese Peer-to-Peer lending market. Finance Research Letters, 2021;39:101559. doi: 10.1016/j.frl.2020.101559

[12] Berger SC, Gleisner F. Emergence of financial intermediaries in electronic markets: The case of online P2P lending. Business Research, 2009;2:39–65. doi: 10.1007/BF03343528

[13] Ding R, Hou W. Retail investor attention and stock liquidity. Journal of International Financial Markets Institutions & Money, 2015;37:12–26. doi: 10.1016/j.intfin.2015.04.001

[14] Takeda F, Wakao T. Google search intensity and its relationship with returns and trading volume of Japanese stocks. Pacific-Basin Finance Journal, 2014;27:1–18. doi: 10.1016/j.pacfin.2014.01.003

[15] Wang XL, Ye Q, Zhao F, Kou Y. Investor sentiment and the Chinese index futures market: Evidence from the internet search. Journal of Futures Markets, 2018;38(4):468–477. doi: 10.1002/fut.21893

[16] Wen FH, Xu LH, Ouyang GD, Kou G. Retail investor attention and stock price crash risk: Evidence from China. International Review of Financial Analysis, 2019;65:101376. doi: 10.1016/j.irfa.2019.101376

[17] Xiao H, Zhang GQ. Public pressure and corporate environmental disclosure–empirical study based on Songhua River Pollution Accident. Accounting research, 2008;5:15–22.

[18] Liu XW, Ye Q. The different impacts of news-driven and self-initiated search volume on stock prices. Information & Management, 2016;53(8):997–1005. doi: 10.1016/j.im.2016.05.009

[19] Li YL, Guo Y, Zhang W. An analysis of impact factors on the loan performance of P2P microfinance market in China. Journal of Finance Research, 2013;7:126–138.

[20] Jiang JL, Liao L, Wang ZW, Zhang XY. Government affiliation and peer-to-peer lending platforms in China. Journal of Empirical Finance, 2021;62:87–106. doi: 10.1016/j.jempfin.2021.02.004

[21] Wang X, Zou L. Research on the policy effect of P2P lending platform trading volume in China: based on the difference of platform type and region. Financial Theory & Practice, 2019;9:49–59.

[22] Wang F, Zhang C. Entry threshold and P2P platform quality: an empirical analysis based on institutional finance. Studies of International Finance, 2019;11:35–44. doi: 10.16475/j.cnki.1006-1029.2019.11.004

[23] Brunnermeier MK, Pedersen LH. Market Liquidity and Funding Liquidity. Review of Financial Studies, 2009;22(6):2201–2238. doi: 10.1093/rfs/hhn098

[24] Fang J, Gozgor G, Lau CKM, Lu Z. The impact of Baidu index sentiment on the volatility of China’s stock markets. Finance Research Letters, 2020;32:101099. doi: 10.1016/j.frl.2019.01.011

[25] Kumar A, Niessen-Ruenzi A, Spalt OG. What’s in a name? Mutual fund flows when managers have foreign-sounding names. Review of Financial Studies, 2015;28(8):2281–2321. doi: 10.1093/rfs/hhv017

[26] Liu S. Investor Sentiment and Stock Market Liquidity. Journal of Behavioral Finance, 2015;16(1):51–67. doi: 10.1080/15427560.2015.1000334

[27] Yang SY, Mo SYK. Social media and news sentiment analysis for advanced investment strategies. Sentiment Analysis Ontology Engineering: An Environment of Computational Intelligence, 2016;639:237–272. doi: 10.1007/978-3-319-30319-2_11

[28] Stein JC. Rational capital budgeting in an irrational world. Journal of Business, 1996;69(4):429–455. doi: 10.1086/209699

[29] Murugan K. A Study on Customer Perception towards Online Marketing in Chengalpattu Town. Emperor Journal of Economics Social Science Research, 2019;1(4):16–23. doi: 10.35338/EJESSR.2019.1404

[30] Kyle AS. Continuous auctions and insider trading. Econometrica, 1985;53(6):1315–1335. doi: 10.2307/1913210

[31] Pagano M. Trading volume and asset liquidity. Quarterly Journal of Economics, 1989;104(2):255–274. doi: 10.2307/2937847

[32] Ferreira MA, Gama PM. Does sovereign debt ratings news spill over to international stock markets? Journal of Banking & Finance, 2007;31(10):3162–3182. doi: 10.1016/j.jbankfin.2006.12.006

[33] Yang XL, Shen HB, Zhu Y. The effect of local bias in investor attention and investor sentiment on stock markets: Evidence from online forum. Journal of Fiancial Research, 2016;12:143–158.

[34] Jiang Q. Research on the efficiency difference and the influencing factors of the volume of P2P loan platform in China. The Journal of Quantitative Technical Economics, 2018;35(6):61–78. doi: 10.13653/j.cnki.jqte.20180608.004

[35] Chen XH, Jin FJ, Lin YH. Research on P2P investment behaviors based on experimental methodology. Science Research Management, 2016;37(11):71. doi: 10.19571/j.cnki.1000-2995.2016.11.009

[36] Lee E, Lee B. Herding behavior in online P2P lending: An empirical investigation. Electronic Commerce Research and Applications, 2012;11(5):495–503. doi: 10.1016/j.elerap.2012.02.001

[37] Diamond DW, Dybvig PH. Bank runs, deposit insurance, and liquidity. Journal of Political Economy, 1983;91(3):401–419. doi: 10.1086/261155

[38] Jacklin CJ, Bhattacharya S. Distinguishing panics and information-based bank runs—welfare and policy implications. Journal of Political Economy, 1988;96(3):568–592. doi: 10.1086/261552

[39] Schmidt L, Timmermann A, Wermers R. Runs on money market mutual funds. American Economic Review, 2016;106(9):2625–2657. doi: 10.1257/aer.20140678

[40] Wang F, Diabat A, Wu L. Supply chain coordination with competing suppliers under price-sensitive stochastic demand. International Journal of Production Economics, 2021;234:108020. doi: 10.1016/j.ijpe.2020.108020

